# Stilbene Induced Inhibition of Androgen Receptor Dimerization: Implications for AR and ARΔLBD-Signalling in Human Prostate Cancer Cells

**DOI:** 10.1371/journal.pone.0098566

**Published:** 2014-06-02

**Authors:** Wolfgang Streicher, Manuel Luedeke, Anca Azoitei, Friedemann Zengerling, Alexander Herweg, Felicitas Genze, Mark G. Schrader, Andres J. Schrader, Marcus V. Cronauer

**Affiliations:** Department of Urology, Ulm University, Ulm, Germany; Innsbruck Medical University, Austria

## Abstract

**Background:**

Advanced castration resistant prostate cancer (CRPC) is often characterized by an increase of C-terminally truncated, constitutively active androgen receptor (AR) variants. Due to the absence of a ligand binding domain located in the AR-C-terminus, these receptor variants (also termed ARΔLBD) are unable to respond to all classical forms of endocrine treatments like surgical/chemical castration and/or application of anti-androgens.

**Methodology:**

In this study we tested the effects of the naturally occurring stilbene resveratrol (RSV) and (E)-4-(2, 6-Difluorostyryl)-N, N-dimethylaniline, a fluorinated dialkylaminostilbene (FIDAS) on AR- and ARΔLBD in prostate cancer cells. The ability of the compounds to modulate transcriptional activity of AR and the ARΔLBD-variant Q640X was shown by reporter gene assays. Expression of endogenous AR and ARΔLBD mRNA and protein levels were determined by qRT-PCR and Western Blot. Nuclear translocation of AR-molecules was analyzed by fluorescence microscopy. AR and ARΔLBD/Q640X homo-/heterodimer formation was assessed by mammalian two hybrid assays. Biological activity of both compounds *in vivo* was demonstrated using a chick chorioallantoic membrane xenograft assay.

**Results:**

The stilbenes RSV and FIDAS were able to significantly diminish AR and Q640X-signalling. Successful inhibition of the Q640X suggests that RSV and FIDAS are not interfering with the AR-ligand binding domain like all currently available anti-hormonal drugs. Repression of AR and Q640X-signalling by RSV and FIDAS in prostate cancer cells was caused by an inhibition of the AR and/or Q640X-dimerization. Although systemic bioavailability of both stilbenes is very low, both compounds were also able to downregulate tumor growth and AR-signalling *in vivo*.

**Conclusion:**

RSV and FIDAS are able to inhibit the dimerization of AR and ARΔLBD molecules suggesting that stilbenes might serve as lead compounds for a novel generation of AR-inhibitors.

## Introduction

Growth and differentiation of benign and malignant prostatic tissue are largely dependent on androgens. Therefore, treatments for patients suffering from locally advanced or metastatic prostate cancer (PC) include the reduction of circulating androgens by surgical or chemical castration as well as the blockade of the androgen receptor (AR) with anti-androgens. Unfortunately the effects of endocrine therapies are only transitory. Although the majority of patients initially responds to first line androgen deprivation therapy (ADT), PC-cells almost invariably acquire the ability to grow and survive in the presence of sub-physiological levels of circulating androgens. This state of the disease, whose onset is characterized by rising titres of serum prostate specific antigen (PSA), is known as castration resistant prostate cancer (CRPC). Although CRPC is resistant to standard endocrine therapies, it is not completely refractory to further hormonal manipulation and AR-signalling still continues to play a pivotal role in the progression of CRPC. The proposed mechanisms that underlie AR function in this clinical setting are quite varied: These include intratumoral androgen synthesis, increased AR-expression and/or stability, deregulation of AR cofactors, aberrant kinase pathway activation as well as AR-mutations broadening AR-ligand specificity [1]. Another putative mechanism enabling CRPC cells to sustain AR-signalling is the expression of constitutively active, C-terminally truncated low molecular weight AR-species lacking the AR-ligand binding domain (LBD) [2]. Due to the absence of a functional LBD these truncated receptors termed ARΔLBDs, are unable to respond to all current forms of therapies targeting the AR-LBD either by the reduction of ligands/androgens or the application of anti-androgens competing with androgens for the AR ligand binding domain. As most ARΔLBDs are usually co-expressed with their normal full length AR counterpart in late stage CRPC, it was hypothesized that ARΔLBDs could affect second generation treatments like abiraterone or enzalutamide. Indeed, there is experimental evidence from several studies that constitutively active ARΔLBDs are limiting the efficacy of abiraterone and or enzalutamide *in vitro* [3]–[5]. These *in vitro* observations could explain, at least in part, the well documented cross-resistance between abiraterone and enzalutamide in the clinical setting [6]–[9].

With the emergence of constitutively active ARΔLBD in late stage CRPC [10]–[12] there is an urgent need for novel compounds, able to inhibit AR-signalling independent of a hormone binding. Resveratrol (RSV; 3, 4', 5-trans-trihydroxystilbene), a stilbene found in a multitude of plants including grapes and peanuts was identified as a potential chemopreventive agent *in vitro* and *in vivo* [13]. In PC RSV has been shown to repress AR-driven gene expression through various mechanisms [14]–[18]. In this study we analyzed the effects of the stilbene RSV and the synthetic fluorinated dialkylaminostilbene ((E)-4-(2, 6-Difluorostyryl)-N, N-dimethylaniline or FIDAS-3 [19] on AR- and ARΔLBD in prostate cancer cells *in vitro* and *in vivo*. The present study shows for the first time that the modulation of AR- as well as ARΔLBD-signalling by synthetic and naturally occurring stilbenes is caused by an inhibition of AR- or ARΔLBD-dimerization.

## Material and Methods

### Plasmids and Chemicals

pSG5-AR, encoding a wild type full length AR (919 amino acids (aa), termed thereafter AR) was supplied by Dr. Z. Culig (Innsbruck, Austria). The expression plasmid pAR-t1EosFP coding for a green fluorescent EosFP-AR-fusion protein was a generous gift from Dr. F. Oswald (Ulm, Germany). The expression plasmids pCruz-ARQ640X and pEGFP-ARQ640X coding for the c-terminally truncated AR-mutant Q640X (aa 1–640) were provided by Dr. J. Céraline (Strasbourg, France). The PSA-reporter plasmid pPSA-61luc was a generous gift of Dr. J. Trapmann (Rotterdam, The Netherlands); the pARE(2x)-luc reporter plasmid pLC0548, created by H. Lebedur was provided by Dr. A. Allera (Bonn, Germany). *Renilla reniformis* luciferase reporter plasmid (pRL-TK) used for the control of transfection efficiency was purchased from Promega (Mannheim, Germany). 3,4',5-trans-trihydroxystilbene or resveratrol (RSV) and its synthetic analog (E)-4-(2,6-Difluorostyryl)-N,N-dimethylaniline, a cell permeable fluorinated N, N-dialkylaminostilbene (FIDAS-3) [19] termed thereafter FIDAS were purchased from Fluka-Sigma-Aldrich, Taufkirchen, Germany and Calbiochem, Merck Biosciences, Darmstadt, Germany. 5α-Androstan-17β-ol-3-one, Dihydrotestosterone (DHT), was provided by Sigma-Aldrich, Taufkirchen, Germany.

### Cell culture

AR-negative PC-3 and the AR-positive LNCaP and 22Rv1 cells were purchased from the American Type Culture Collection (Manassas, VA, USA). The CRPC cell line LNCaP C4-2 originally described by Wu et al. [20], was provided by Prof. Sven Reske, (Ulm, Germany). RPMI-1640, phosphate buffered saline and penicillin/streptomycin-solution were products of PAA Laboratories (Linz, Austria). Fetal bovine serum (FBS) and steroid-free dextran-charcoal-treated FBS (FBSdcc) were obtained from BioWest (Nuaille, France). Cell culture plastic ware was purchased from Sarstedt (Nürmbrecht, Germany). LNCaP, 22Rv1 and PC-3 cells were routinely cultured in RPMI-1640, 1% penicillin/streptomycin (v/v), 10% FBS (v/v) whereas LNCaP C4-2 were routinely grown in RPMI-1640 supplemented with 1% penicillin/streptomycin (v/v) and 10% FBSdcc (v/v). During experiments, cells were maintained in RPMI-1640 with 5% FBSdcc (v/v) and antibiotics in the presence/absence of DHT and RSV or FIDAS.

### Nuclear translocation assay

PC-3-cells were seeded in 24-well plates and grown in the absence of DHT for 24 hours. Subsequently, cells were transfected with pAR-t1EosFP and pEGFP-ARQ640X, respectively. After 24 hours pAR-t1EosFP transfected cells were treated with ethanol (solvent control) or 5 nM DHT for 2 hours in presence/absence of RSV (100 µM) or FIDAS (50 µM). pEGFP-ARQ640X transfected cells expressing constitutively active GFP-tagged Q640X did not need an androgenic stimulus and were treated with RSV and FIDAS only. Subsequently, nuclear/cytoplasmic fluorescence was determined in the cells using fluorescence microscopy.

### Western Blot

Prostate cancer cells were lysed in a buffer containing 10 mM Tris/HCl pH 7.6, 5 mM EDTA, 50 mM NaCl, 50 mM NaF and 1% Triton X100 (v/v). Cell nuclei and debris were removed by centrifugation (3 min, 10,000 x g), and the supernatant was tested for protein concentration using the BCA-method (Sigma Aldrich, Taufkirchen, Germany). Cell extracts were electrophoresed through a 10% SDS-PAGE gel (Novex, Carlsbad, CA, USA) and electroblotted onto a nitrocellulose or PVDF (Pall, Bad Kreuznach, Germany and Merck Millipore, Darmstadt, Germany). AR protein was detected by monoclonal mouse antibody AR441 (Dako, Hamburg, Germany, 1∶2000), detection of β-actin with mouse monoclonal Anti-β-Actin (Sigma Aldrich, Taufkirchen, Germany, 1∶2000) served as loading control. Immunoreactive bands were localized using horseradish peroxidase-labeld goat anti-mouse antibody (Santa Cruz Biotechnology, Santa Cruz, CA; 1∶2000-1∶10000). Immunoreactive complexes were visualized by direct detection of chemoluminescene using the Fusion FX7 Imaging and Gel documentation system and FUSION-CAPT software (Vilber Lourmat, Marne-la-Vallee, France).

### Reportergene assays

AR-signalling was analyzed by reportergene assays. Therefore the AR-negative PC-3, cells were transiently co-transfected in 24-well plates with AR and/or Q640X-expression plasmids (pSG5-AR; pCRUZ-ARQ640X) and different reporter gene constructs (pPSA-61luc, pARE(2x)-luc) using Polyfect (Qiagen, Hilden, Germany). pRL-tk-LUC was co-transfected as an internal control for transfection efficiency. 24 hours after transfection, cells were treated with/without 5 nM DHT in the presence/absence of the stilbenes RSV (50 and 100 µM) and FIDAS (50 µM). After 24 hours reportergene activity was measured using the Dual-Luciferase Reporter Assay (Promega GmbH, Mannheim, Germany) according to the manufactures instructions. Within this time frame RSV as well as FIDAS did not exhibit a significant *in vitro* toxicicity (see Figure S1).

### Mammalian two Hybrid assay (M2H)

Dimerization of AR and/or Q640X was analyzed using the CheckMate Mammalian Two-Hybrid (M2H) System (Promega, Mannheim, Germany) according to the manufactures instructions. The CheckMate M2H contains 2 expression vectors termed pBIND and pACT. The pBIND vector expresses a yeast GAL4 DNA-binding domain upstream of a multiple cloning region and a SV40-controlled *Renilla reniformis* luciferase for transfection control. The pACT-vector contains the herpes simplex VP16 activation domain upstream of a multiple cloning region. The genetic information coding for the interactive proteins of interest (AR, Q640X) were subsequently cloned into the pBIND and pACT–vectors to generate fusion proteins with the DNA-binding domain of GAL4 and the activation domain of VP16. The pGAL4 and pVP16 fusion constructs (pAR-VP16/ACT, pAR-GAL4/BIND, pQ640X-VP16/ACT, pQ640X-GAL4/BIND) were transfected along with pG4.31-luc which encodes a GAL4-dependent firefly luciferase reporter. Dimerization of two chimeric fusion proteins like AR-VP16/ACT and AR-GAL4/BIND (AR homodimer), Q640X-VP16/ACT and Q640X-GAL4/BIND (Q640X homodimer) or AR-VP16/ACT and Q640X-GAL4/BIND (AR/Q640X-heterodimer) promoted the activation of the M2H reporter pG4.31-luc with subsequent expression of firefly luciferase. Amounts of firefly luciferase and Renilla luciferase were subsequently detected using the Dual-Luciferase Reporter Assay System.

### RNA isolation and quantitative RT-PCR

Total RNA was isolated from 22RV1, LNCaP and LNCaP C4.2 cells using the RNeasy Mini kit (Qiagen, Hilden, Germany) according to the manufactures instructions. The mRNA levels of full length AR and the AR splice variant AR-V7 were determined by the relative quantification method (ΔΔCt) using glucose 6-phosphate dehydrogenase (G6PD) as housekeeping gene. The quantification was performed with TaqMan probes along with the QuantiFast Multiplex RT-PCR + R Kit (Qiagen, Hilden, Germany) on a ViiA 7 Real-Time PCR (Applied Biosystems, Life Technologies, Carlsbad, USA). Primers were designed to span exon/exon boundaries in order to avoid signals from residual genomic copies of the gene. Primers and MGB-probes (sequences listed in Table 1) were purchased from biomers.net, Ulm, Germany and Applied Biosystems, Life Technologies, Carlsbad, USA, respectively. Cycling conditions were as follows: 50°C for 20 min and 95 °C for 5 min (initial cDNA synthesis and hot start activation), followed by 40 cycles at 94°C for 15 sec and 60°C for 60 sec.

**Table 1:** Primers and Probes for qRT-PCR.

Gene	forward primer (5′-3′)	reverse primer (5′-3′)	Taqman-probe (5′-3′)
**AR**	GGACTCCGTGCAGCCTATT	GGAAAGTCCACGCTCACCA,	6FAM-CCTGCTAATCAAGTCACAC-MGB-NFQ
**AR-V7**	GGATGACTCTGGGAGAAAAATTC	CTTTCTTCAGGGTCTGGTCATT	6FAM-CAATTGCAAGCATCTCA-MGB-NFQ,
**G6PD**	CCGGGCATGTTCTTCAA	AGGGAGCTTCACGTTCTTGTAT	VIC-TCCAGCTCCGACTCC-MGB-NFQ

### CAM-Assay

In order to analyze the effects of FIDAS *in vivo* we used a modified chick chorioallantoic membrane (CAM) assay as an animal substitute model [21]. Therefore, shells of fertilized chicken eggs were opened on day 8 and silicon rings (diameter 5 mm) were applied onto the CAM. One million cells were seeded into the ring in 20 µl 50% Matrigel (v/v) (BD Biosciences, Heidelberg, Germany) dissolved in serum and antibiotics free RPMI-1640. Tumor grafts were allowed to grow for 48 hours. In contrast to the classical CAM-assay, RSV and FIDAS were not applied topically. Instead 50 µl of the compounds [RSV 100 µM; FIDAS 50 µM] were injected into a CAM-vein [21], [22], allowing the systemic spread of the compound in the chick embryo-CAM system for 48 hours. Subsequently tumor tissues were fixed, paraffin embedded and serially sectioned. The effects of RSV and FIDAS on proliferation and AR-signalling were monitored by immunohistochemistry of KI67 and PSA using monoclonal mouse anti-human Ki-67 antigen, Clone MIB-1 and mouse anti-human prostate.specific antigen, clone ER-PR8, DAKO Diagnostica, Hamburg, Germany [21], [22].

### Statistical Analysis

Data are reported as means ± standard deviation. Analysis was performed with Student's T-test (two tailed for independent samples) with p<0.05 considered as significant.

## Results

### RSV and FIDAS inhibit signalling of endogenous AR in LNCaP and 22Rv1 cells

Various natural and synthetic stilbenes were recently shown to exhibit strong chemopreventive and anti-proliferative effects in experimental PC [23]. Moreover, RSV has repeatedly been shown to down-regulate AR-signalling in PC cells [14], [16], [17], [24]. The following experiments were performed to demonstrate the ability of the synthetic fluorinated RSV-analog (E)-4-(2, 6-Difluorostyryl)-N, N-dimethylaniline (FIDAS-3, termed thereafter FIDAS) [19] to modulate AR-signalling in the PC cell lines (Table 2). In parallel treatment with the naturally occurring RSV served as control. Due to the relative low aqueous solubility of FIDAS-agents [19], [25] the maximal concentration used in the experiments was 50 µM. For RSV-experiments the recommended concentration should not exceed 150 µM [17].

**Table 2:** RSV and FIDAS down-regulate activity of AR-dependent reporter-gene activity in LNCaP and 22Rv1 cells.

**RSV [ µM]**	**0**	**50**	**100**	**0**
**FIDAS [ µM]**	**0**	**0**	**0**	**50**
**LNCaP**	100	47±23*	33±13**	35±9**
**22Rv1**	100	39±27^**^	9±7**	18±12**

AR-positive LNCaP and 22Rv1 cells were transfected with the reporter-gene plasmids pARE(2x)-luc and pRT-TK, as described in Material and Methods. Subsequently cells were incubated with RSV or FIDAS in presence/absence of 5 nM DHT for 24 hours. AR-reporter-gene activity was determined using the Dual-Luciferase Reporter Assay System (Promega) according to the manufactures instructions. Results are expressed in % (AR_DHT stimulated_/AR_basal activity_) which was set at 100% for RSV or FIDAS untreated cells (controls). Each value represents the mean of at least 3 independent experiments ± standard deviation, (p-values compared to corresponding DHT-treated control *p<0.01, **p<0.05)

Effects of FIDAS on AR-signalling were analyzed using an AR-specific reporter gene assay. Full AR activity was achieved following stimulation of the cells with 5 nM DHT (stilbene untreated controls set at 100%). In LNCaP and 22Rv1 cells treated with FIDAS (50 µM) transcriptional activity was diminished to 35% and 18%, respectively. RSV-controls at the same concentration reduced AR transcriptional activity of DHT-stimulated LNCaP and 22Rv1 cells to 47% and 39%, respectively. When treated with the maximal concentration of 100 µM RSV transcriptional activity of the AR was reduced to 33% in LNCaP and 9% in 22Rv1 cells.

### Effect of RSV and FIDAS on AR and ARΔLBD mRNA levels in human prostate cancer cell lines

Several studies suggest that the predominant mechanism enabling RSV to inhibit AR-signalling is through a down regulation of AR-mRNA [17], [24]. However, these results are discussed controversially [18], [26], [27]. Unfortunately, all studies analyzing AR mRNA-levels in PC cells were done in the hormone sensitive LNCaP cells, only. In consequence, we tested the effects RSV and FIDAS in LNCaP, LNCaP C4-2 (a castration resistant LNCaP subline) and in the AR positive CRPC cell line 22Rv1, known also to express high levels of ARΔLBDs [28].

The ability of the stilbenes RSV and FIDAS to modulate basal endogenous AR or ARΔLBD mRNA-levels were studied by qRT-PCR. As endogenous ARΔLBD we analyzed the expression of the splicing variant AR-V7 (also termed AR3), a prototypical ARΔLBD [2], [28], [29]. Whereas LNCaP and LNCaP C4-2 express relatively low levels of AR-V7, the CRPC cell line 22Rv1 has been shown to produce very high levels of AR-V7 mRNA and protein [28]. In comparison to full length AR (mRNA set at 100% for each cell line) the expression levels of the AR-V7 determined by qRT-PCR were approximately 3% AR-V7 in LNCaP and LNCaP C4-2 and 250% AR-V7 in 22Rv1 (data not shown).

In agreement with previous findings [17], [24] RSV was able to downregulate AR-mRNA in LNCaP and LNCaP C4-2 cells (Table 3). Downregulation was already detectable at concentration of 10 µM. The ability of RSV to downregulate AR-mRNA was more pronounced in the castration resistant LNCaP C4-2 cells as compared to the hormone sensitive, parental LNCaP cells (basal AR-mRNA levels in LNCaP: untreated 100%; 10 µM RSV 94%, 100 µM RSV 73% versus LNCaP C4-2: untreated 100%; 10 µM 51%, 100 µM RSV 34%). This tendency could also be confirmed for AR-V7 (basal AR-V7 mRNA levels in LNCaP: untreated 100%; 10 µM RSV 65%, 100 µM RSV 51% versus LNCaP C 4–2: untreated 100%; 10 µM 37%, 100 µM RSV 39%). In general, RSV also decreased AR and AR-V7 mRNA levels in 22Rv1 cells (basal AR-mRNA levels in 22Rv1 cells: untreated 100%; 10 µM RSV 74%, 100 µM RSV 116% and basal AR-V7 mRNA levels in 22Rv1 cells: untreated 100%; 10 µM 70%, 100 µM RSV 73%). For unknown reasons however, the highest concentration of 100 µM RSV induced a slight but persistent increase of AR mRNA (116%) in 22Rv1 cells.

**Table 3:** Effects of RSV and FIDAS on AR- and AR-V7-mRNA-expression in prostate cancer cell lines.

	LNCaP		LNCaP C4.2		22Rv1	
**RSV [ µM]**	**AR**	**AR-V7**	**AR**	**AR-V7**	**AR**	**AR-V7**
**ctrl**	100±0	100±0	100±0	100±0	100±0	100±0
**10**	94±25	65±13 **	51±17 **	37±15 **	74±11 **	70±8 **
**100**	73±23 *	51±19 **	34±16 **	39±32 **	116±6 **	73±10 **
**FIDAS [ µM]**	**AR**	**AR-V7**	**AR**	**AR-V7**	**AR**	**AR-V7**
**ctrl**	100±0	100±0	100±0	100±0	100±0	100±0
**10**	87±15	60±5 *	77±3 *	89±4	91±8	96±4
**50**	62±7 *	44±6 **	36±4 **	55±8 *	94±21	108±6

RNA isolation and qRT-PCR were performed as described in Material and Methods. Data are expressed in % of RSV or FIDAS untreated controls which were at 100% (ctrl). Each value represents the mean of at least 3 independent experiments ± standard deviation (p-values compared to untreated controls, *p<0.05 and **p<0.01)

In LNCaP and LNCaP C4-2 cells FIDAS also induced a dose dependent decrease of AR and AR-V7 mRNA (Table 3). The FIDAS induced decrease of AR mRNA was more pronounced in the castration resistant LNCaP C4-2 than in LNCaP (basal AR-mRNA levels in LNCaP: untreated 100%; 10 µM FIDAS 87%, 50 µM FIDAS 62% versus LNCaP C4-2: untreated 100%; 10 µM FIDAS 77%, 100 µM RSV 36%). FIDAS also reduced the AR-V7 mRNA levels in both cell lines, although this time AR-V7 mRNA levels were more affected in the parental LNCaP cells (basal AR-V7 mRNA levels in LNCaP: untreated 100%; 10 µM FIDAS 60%, 50 µM FIDAS 44% versus LNCaP C 4-2: untreated 100%; 10 µM FIDAS 89%, 100 µM RSV 55%). To our surprise FIDAS was unable to significantly decrease AR and AR-V7 mRNA levels in 22Rv1 cells.

### Effect of RSV and FIDAS on AR and ARΔLBD protein levels in human prostate cancer cell lines

The ability of high RSV concentrations to increase AR mRNA levels in LNCaP and the general inability of FIDAS to reduce AR and AR-V7 mRNA levels in 22Rv1, although the compounds diminished AR-signalling in both cell lines, prompted us to analyze the effects of RSV and FIDAS on AR-protein levels in LNCaP and 22Rv1 cells. This approach is furthermore supported by a report of Harada et al. showing that RSV represses AR target gene expression, at least partially, by regulating AR-degradation [14].

**Figure 1:**
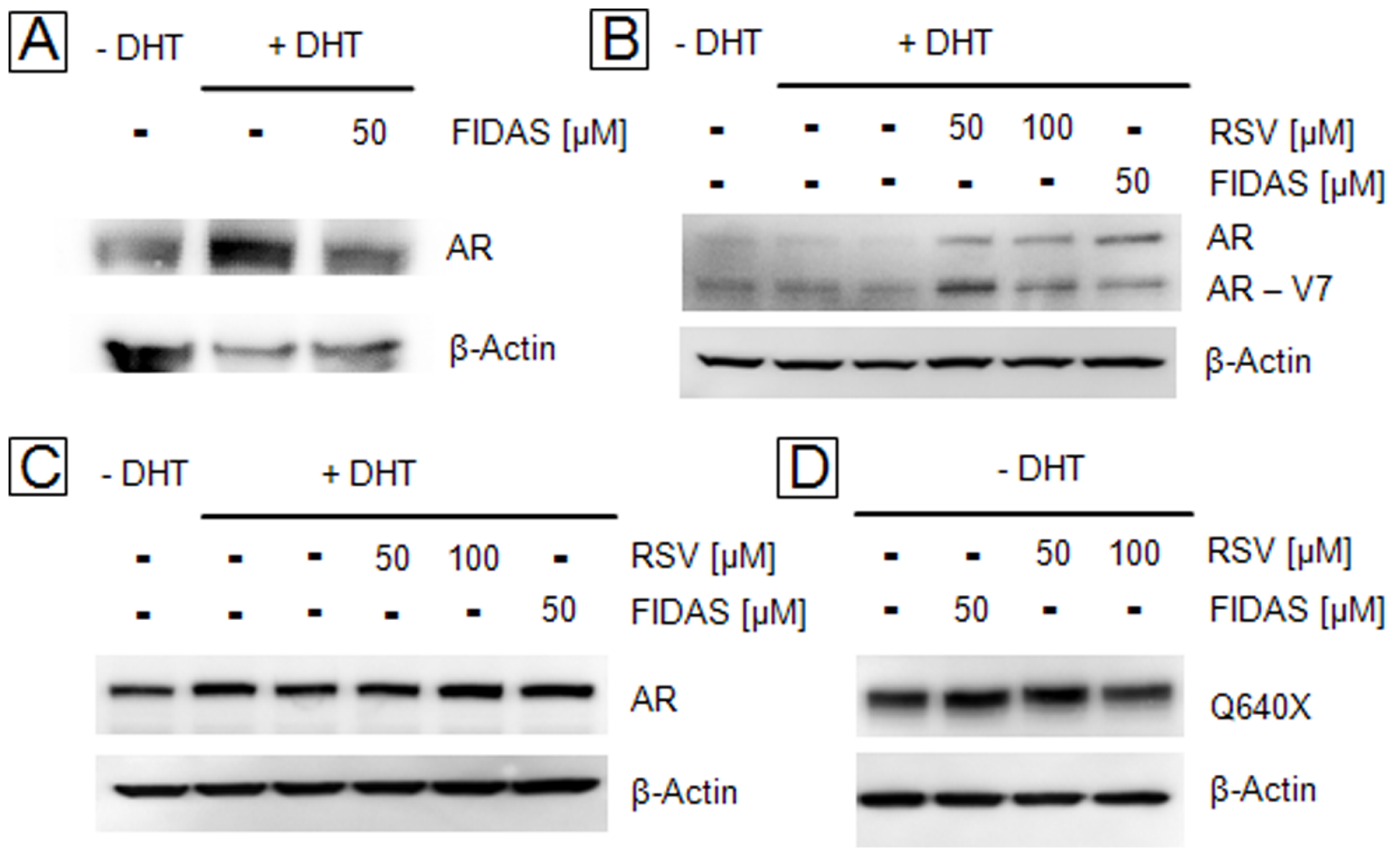
Regulation of AR- and ARΔLBD-protein by RSV and FIDAS in human PC cell lines. Prostate cancer cells were grown in presence/absence of DHT and incubated with RSV or FIDAS for 24 hours. Subsequently, intracellular AR or ARΔLBD-levels (AR-V7, Q640X) were determined by Western Blotting as described in Material and Methods: (**A**) LNCaP, (**B**) 22Rv1, (**C**) PC-3 transfected with full length AR, (**D**) PC-3 transfected with Q640X.

As seen in Figure 1A, AR-protein levels were diminished in LNCaP cells following FIDAS-treatment, a finding that is in agreement with the mRNA-data. By contrast, in 22Rv1 cells, both compounds increased rather than decreased AR- and AR-V7 protein levels, irrespective of previous mRNA-levels (Figure 1B). In PC-3 cells transiently transfected with AR or Q640X expression plasmids under control of a constitutive SV40 or CMV promoter, the protein levels of both AR-variants remained unchanged following RSV or FIDAS treatment (Figure 1C, D) suggesting that AR-protein levels and/or AR-stability are not generally affected in all PC cells.

In consequence, in order to analyze the direct effects of FIDAS and RSV on AR and ARΔLBD-signalling without interference from changes in AR mRNA levels, the following experiments were performed in PC-3 cells transiently transfected with AR and/or Q640X

### RSV and FIDAS inhibit the transcriptional activity of the C-terminally truncated constitutively active Q640X in PC-3 cells

**Figure 2:**
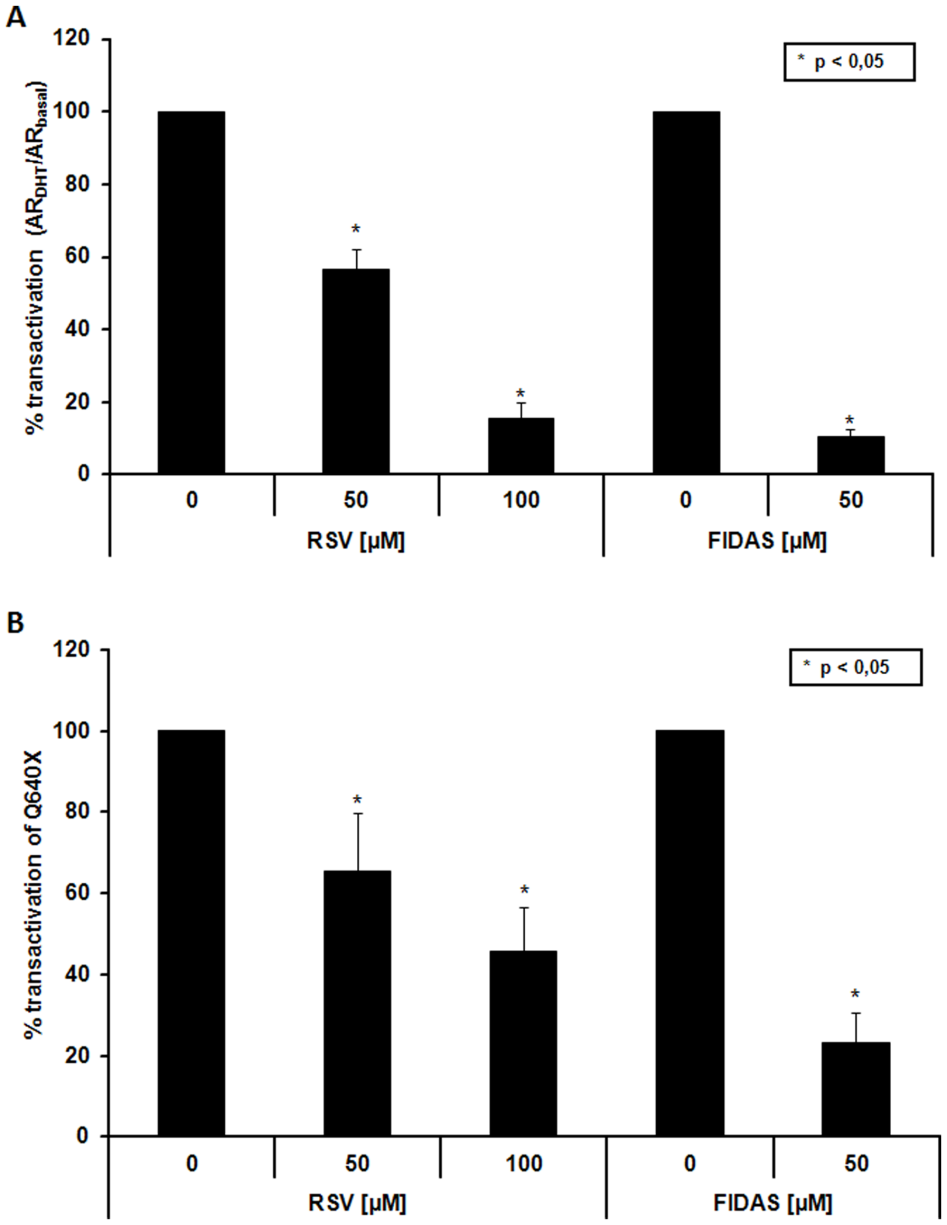
Effects of FIDAS and RSV on AR and Q640X signalling. PC-3 cells were transiently co-transfected with AR or Q640X, the pARE(2x)-luc reporter and the pRL-TK control plasmid as described in Material and Methods. (**A**) PC-3 cells transfected with AR were grown with/without 5 nM DHT in the presence/absence of RSV or FIDAS for 24 hours. Data are expressed as % transactivation of DHT stimulated AR ( =  % activity AR_DHT_/AR_basal_) which was set at 100%; *p<0.05. (**B**) PC-3 cells transfected with constitutively active Q640X were treated with RSV or FIDAS for 24 hours. Data are expressed as % transactivation of Q640X which was set at 100%, *p<0.05).

In order to analyze the effects of RSV and FIDAS on ARΔLBD-signalling, AR-negative PC-3 were co-transfected with the constitutively active ARΔLBD-variant Q640X and an AR-dependent reportergene pARE(2x)-luc. Co-transfected pRL-tk-LUC served as transfection control. In parallel, experiments were performed with full length AR in presence/absence of DHT. Both RSV and its fluorinated counterpart FIDAS were able to significantly down-regulate the transcriptional activities of AR (Figure 2A) and Q640X (Figure 2B). When comparing RSV to FIDAS, the latter was the more potent inhibitor of AR and Q640X signalling (100 µM RSV: AR-activity 16% and Q640X-activity 46% - 50 µM FIDAS: AR-activity 10% and Q640X-activity 23%). The fact that both compounds were able to inhibit the LBD-lacking Q640X clearly shows that RSV like FIDAS does not attenuate AR-function through binding to the AR-ligand binding domain.

### RSV and FIDAS inhibit signalling of AR/ARLΔBD-heterodimers

**Figure 3:**
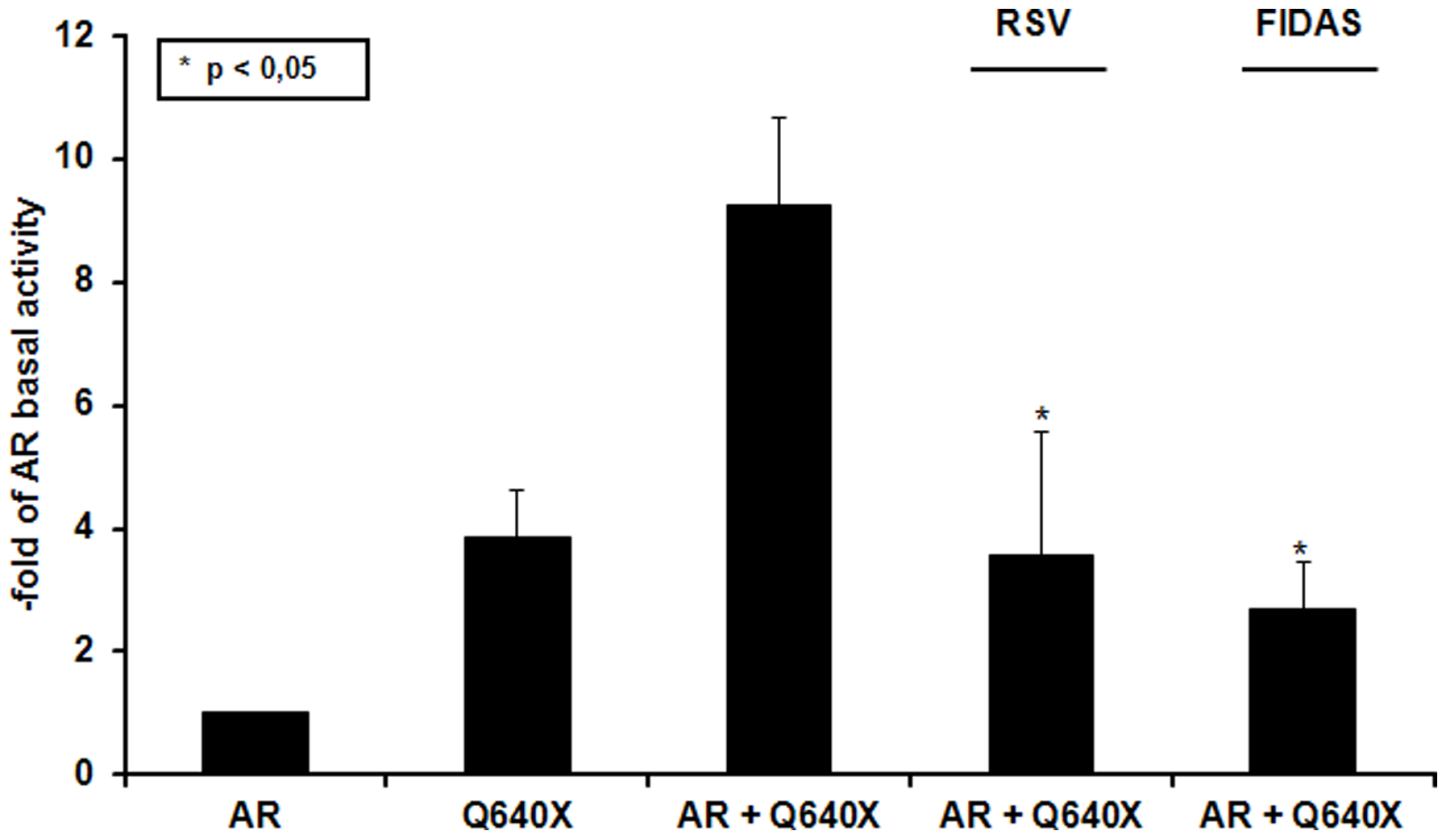
Effects of RSV and FIDAS on AR/Q640X-signalling under androgen deprived conditions. PC-3 cells were transiently transfected with AR, Q640X or AR+Q640X, together with pPSA-61luc and pRL-TK reporter plasmids as described in Material and Methods. Cells expressing Q640X and/or AR were grown for 24 hours in presence/absence of RSV (100 µM) or FIDAS (50 µM). Data are expressed in fold AR basal activity which was set to 1. As depicted, RSV and FIDAS significantly decreased PSA-promoter mediated reporter gene activity in Q640X and AR co-expressing cells, *p>0.05.

In the absence of androgens, co-expression of a full length AR with an ARΔLBD like Q640X or AR^v567es^ was shown to form transcriptionally active heterodimers [30], [31]. As seen in Figure 3 Q640X did only weakly activate the PSA promoter driven reporter plasmid Q640X (4-fold basal activity of DHT-untreated AR which was set at 1). Interestingly a co-expression of AR with Q640X in PC-3 cells grown in the absence of androgens led to a synergistic transactivation of the PSA-promoter (9-fold basal activity of the AR) suggesting the formation of AR/Q640X heterodimers. As depicted in Figure 3 both RSV and FIDAS significantly inhibited PSA-promoter dependent reporter gene activity induced AR/Q640X-heterodimers. Maximal PSA-reporter gene activity induced by AR/Q640X was reduced by 61% and 71% following RSV (100 µM) or FIDAS (50 µM) treatment.

### RSV and FIDAS do not modulate nuclear localization of AR and Q640X

**Figure 4:**
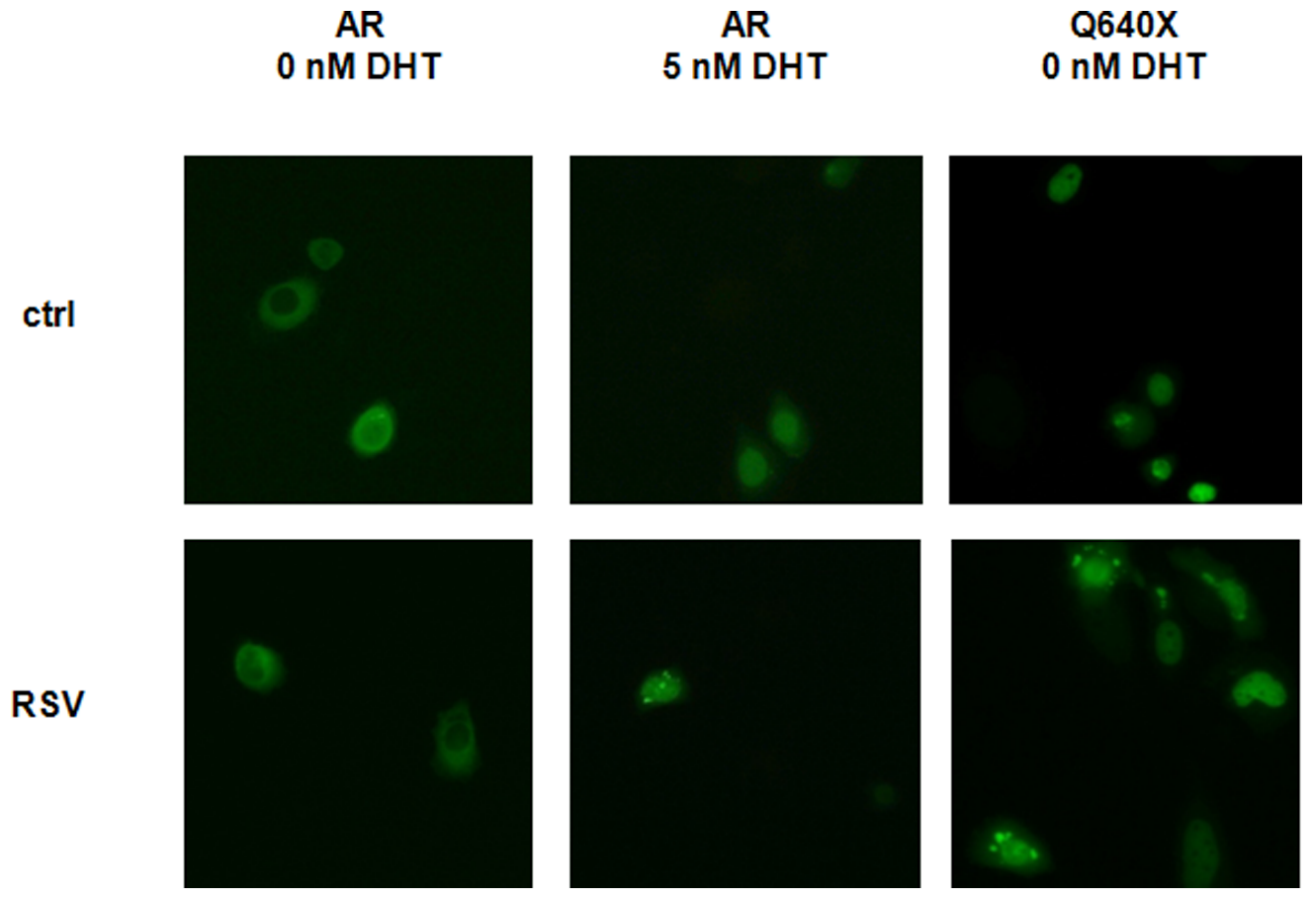
RSV and FIDAS do not inhibit nuclear translocation of AR and Q640X. PC-3 cells were transiently transfected with expression plasmids coding for green fluorescent AR-EosFP or Q640X-EGFP fusion proteins as described in Material and Methods. Subsequently cells were treated with/without 5 nM DHT in presence/absence of 100 µM RSV for 120 minutes. Nuclear localization of fluorescent AR or Q640X proteins was analyzed by fluorescence microscopy.

Based on our results that AR-expression and AR-stability cannot fully account for RSV or FIDAS-mediated inhibition of AR-signalling, we analyzed the effects of both stilbenes on the nuclear translocation of AR and Q640X. Previous studies analyzing the effects of RSV on the nuclear translocation of AR remained controversial [16], [17]. In order to test the effects of RSV and FIDAS on the nuclear import of AR and ARΔLBD we transfected AR-negative PC-3 cells with EosFP-tagged AR or EGFP-tagged Q640X. Nuclear localization of the green fluorescent fusion proteins was subsequently analyzed by fluorescence microscopy [30]. As depicted in Figure 4 nuclear translocation AR or Q640X was not significantly affected by RSV. Treatment with FIDAS yielded similar results (Table 4).

**Table 4:** Effects of RSV and FIDAS on the nuclear translocation of AR and Q640X.

	AR			Q640X	
**DHT [5 nM]**	**−**	**+**	**+**	**−**	**−**
**RSV [ µM]**	**0**	**0**	**100**	**0**	**100**
**% nuclear localization**	4±3	82±11	73±8	100±1	99±1
**DHT [5 nM]**	**−**	**+**	**+**	**−**	**−**
**FIDAS [ µM]**	**0**	**0**	**50**	**0**	**50**
**% nuclear localization**	6±5	88±4	86±7	99±1	99±0

Prostate cancer cells (PC-3) were transfected with expression plasmids coding for green fluorescent AR-EosFP or Q640X-EGFP fusion proteins. Nuclear localization of AR or Q640X fusion proteins was analyzed by fluorescence microscopy. Data present the percentage of fluorescent cells exhibiting a predominantly nuclear fluorescence.

### RSV and FIDAS inhibit the dimerization of AR and Q640X-receptor molecules

In order to generate genomic signals the AR has to dimerize with another AR-molecule in the nucleus [32]. The observation that RSV and FIDAS were able to inhibit signalling induced by AR/AR and Q640X/Q640X–homodimers (Figure 2) as well as by AR/Q640X-heterodimers (Figure 3) prompted us to analyze the effects of both compounds on the dimerization of AR and/or Q640X using a specially designed M2H Assay [30].

The M2H is a powerful tool to identify protein-protein interactions in living cells. In contrast to conventional M2H procedures that are mainly using isolated functional domains of proteins to analyze protein-protein interaction, our experimental setup is based on fully active transcription factors fused to a DNA-binding GAL4-binding domain or a VP16-activation domain (AR-GAL-4/BIND, AR-VP-16/ACT; Q640X-GAL4/BIND, Q640X-VP16/ACT). Upon dimerization the chimeric GAL4/BIND and VP16/ACT receptor fusion proteins are able to promote the activation of the M2H reporter controlled by a GAL4-responsive promoter. However, a M2H-approach for the analysis of dimerization processes using fully active transcription factors like AR or Q640X requires extensive control experiments (see supporting information). In contrast to the nucleosolic formation of AR-dimers following androgenic stimuli [32] the dimerization process of ARΔLBDs is thought to be DNA driven taking only place in the presence of an androgen response element (ARE). Moreover, AR and Q640X-transcription factor dimers are able to induce transactivation without a VP16 activation domain. As AR-GAL4/BIND or Q640X-GAL/BIND are able to bind the M2H reporter gene construct via their GAL4-binding site one has to control that they are unable to activate the M2H-reporter without the need of a corresponding AR-VP16/ACT or Q640X-VP16/ACT dimerization partner. In the presence of androgens, the AR-GAL4/BIND fusion protein was unable to induce a significant M2H-reporter gene activity when of AR-VP16/ACT was not coexpressed (Figure S1). Similar results were found for the constitutively active Q640X-GAL4/BIND (Figure S1) suggesting that the M2H is an adequate tool for the analysis of AR or Q640X-dimerization.

**Figure 5:**
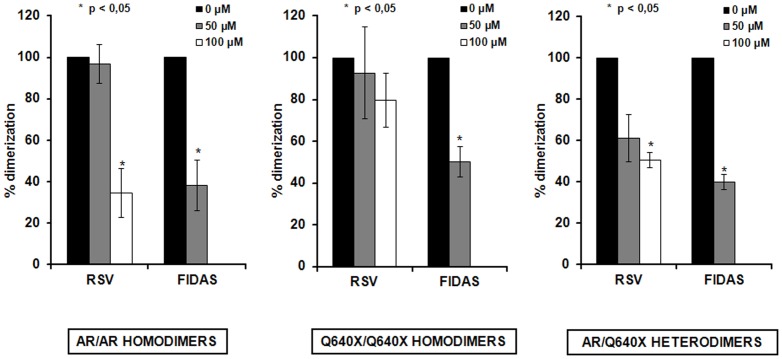
Effects of RSV and FIDAS on the dimerization of AR and Q640X. Formation of AR and Q640X homo/hetero-dimers was analyzed 24 hours after RSV or FIDAS treatment using a M2H as described in Material and Methods. Within this time frame RSV as well as FIDAS did not exhibit a significant *in vitro* toxicicity (see Table S1). ***AR/AR dimers***: Formation of AR/AR homodimers was analyzed in PC-3 cells grown under androgenic stimuli (5 nM DHT) in presence/absence of RSV or FIDAS (FIDAS/RSV untreated + DHT  = 100%). ***AR/Q640X and Q640X/Q640X dimers***: Formation of AR/Q640X heterodimers (AR-VP16/ACT and Q640X-GAL4/BIND) or Q640X/Q640X homodimers was analyzed in the absence of DHT in RSV/FIDAS treated/untreated PC-3 cells (FIDAS/RSV untreated was set at 100%), *p<0.05.

As can be seen in Figure 5, both RSV and FIDAS were able to inhibit the formation of AR/AR-homodimers in the presence of 5 nM DHT. A dramatic and statistically significant decrease in AR-dimerization was detectable at RSV and FIDAS concentrations of 100 µM and 50 µM, respectively (%AR-dimerization in stilbene untreated cells 100%, %AR-dimerization at 100 µM RSV 34%; %AR-dimerization at 50 µM FIDAS 38%). As Q640X is constitutively active, all experiments with this ARΔLBD were performed in the absence of DHT. Although RSV showed a tendency do down regulate Q640X-dimerization, inhibition of Q640X/Q640X-homodimer formation was only significantly affected by FIDAS (%Q640X-dimerization in stilbene untreated cells 100%, %Q640X-dimerization at 100 µM RSV 80%; %Q640X- dimerization at 50 µM FIDAS 50%) (Figure 5). Most interestingly RSV and FIDAS were able to significantly decrease AR/Q640X heterodimerization, recently observed to occur in the absence of androgens [30]. Heterodimerization was inhibited at RSV and FIDAS concentrations of 100 µM and 50 µM, respectively. Transfection of PC-3 cells with control vectors (pBIND-ID and pACT-MyoD, Promega) that encode and express two proteins known to interact *in vivo* showed that ID/MyoD-dimerization remained unaffected by RSV and FIDAS in the M2H, the latter suggesting that the effects of stilbenes on AR- and Q640X-dimerization are specific (data not shown).

### Effects of RSV and FIDAS on AR-positive prostate cancer cells *in vivo*

**Figure 6:**
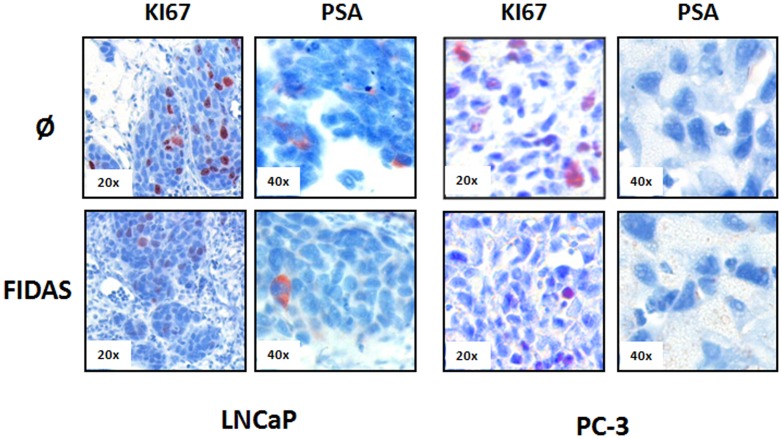
Effects FIDAS on prostate cancer micro-tumors growing on the CAM. CAM assays were performed with PC-3 (AR negative) and LNCaP (AR-positive) as described in Material and Methods. Proliferation of PC cells was determined by nuclear staining of KI67. AR-activity was analyzed by PSA-staining.

The poor aqueous solubility of RSV and RSV-derivates like FIDAS [19], [33] limit their bioavailability. In order to test the efficacy of RSV and FIDAS as potential therapeutic agents, we extended our studies to a modified CAM xenograft model. Therefore 50 µl of the compounds solubilised in a physiological salt solution at a final concentration of 100 µM and 50 µM (maximal *in vitro* test concentrations) were administered intravenously (CAM-vein) allowing the systemic spread of the substances in the chick embryo-CAM system [21]. As seen in Figure 6 RSV and FIDAS inhibited the proliferation of PC cells *in vivo*. The downregulation of proliferation as reflected by KI67 was more pronounced in the AR-positive LNCaP than in the AR-negative PC-3 (proliferation rate, KI67 Table 5): LNCaP, untreated 100%, 100 µM RSV 50+/-33%, 50 µM FIDAS 60+/−19% and PC-3, untreated 100%, 100 µM RSV 86+/−7%, 50 µM FIDAS 62+/−38%). Additionally AR-activity was inhibited by both stilbenes as depicted by a down regulation of PSA in the tumor xenografts (PSA expressing LNCaP cells, untreated 100%, 100 µM RSV 27+/−24%, 50 µM FIDAS 64+/−29%). Our results show that even low systemic concentrations following intravenous injection are sufficient to impair proliferation as well as AR-signalling

**Table 5:** Effects of RSV and FIDAS on on PCa cells *in vivo*.

		ctrl	RSV [100 µM]	FIDAS [50 µM]
**KI67**	**LNCaP**	100	50%±33	60%±19
**KI67**	**PC-3**	100	86%±7	62%±38
**PSA**	**LNCaP**	100	27%±24	64%±29

PC-3 and LNCaP cells were seeded onto the CAM to form tumors as described Material and Methods. Tumor grafts were allowed to grow for 48 hours. Subsequently, 50 µl of RSV (100 µM) or FIDAS (50 µM) were injected into a CAM-vein allowing the systemic spread of the compound. After 48 hours tumor xenografts were fixed, paraffin embedded and serially sectioned. Microtumors were stained for KI67 and PSA. Data are expressed in % of KI67 or PSA positive cells, grown in the absence of RSV or FIDAS ± standard deviation of at least 3 independent experiments (ctrl  =  RSV/FIDAS untreated  = 100%).

## Discussion

Although the majority of patients initially respond well to ADT, PC almost invariably recurs in a form that is no longer susceptible to classical endocrine therapies. This state of the disease, whose onset is characterized by rising titres of serum PSA at castrate levels of circulating androgens, is known as CRPC. Interestingly, the AR still plays a central role at this stage of the disease. The mechanisms allowing CRPC cells to activate the AR despite subphysiological levels of circulating androgens include intratumoral androgen synthesis (steroidogenic pathway), deregulation of steroid receptor cofactors and/or increase of intracellular AR-levels (hypersensitive pathway), point mutations broadening AR-ligand specificity (promiscuous pathway) and AR activation through tyrosine kinase-dependent mechanisms (outlaw pathway) [1], [34]. Albeit CRPC cells are resistant to standard endocrine therapies, they are not completely refractory to further hormonal manipulation as evidenced by successful treatments with enzalutamide or abiraterone targeting the androgen/AR-axis. Unfortunately, the efficacy of second line hormonal treatments is only transitory: A further mechanism enabling especially CRPC cells to sustain the activation of AR-dependent genes, is the expression of constitutively active, C-terminally truncated AR-species lacking their ligand binding domain (ARΔLBD-pathway) [2], [28], [30], [31]. Due to the absence of a functional ligand binding domain (LBD), these AR-variants termed ARΔLBD, are unable to respond to all current forms ADT. This phenomenon highlights the importance of novel compounds able to inhibit AR as well as ARΔLBD function.

The AR is a ligand binding transcription factor of the steroid hormone receptor superfamily. In the absence of androgens, the AR is predominantly located in the cytoplasm. Upon androgen binding the AR-protein translocates into the nucleus where it dimerizes with another AR-molecule. The AR-dimer subsequently binds to androgen response elements present in the cis-regulatory regions of target genes. Early functional studies showed that a deletion of the AR-LBD situated in the C-terminus of the AR, results in constitutive activation of the receptor molecule [35]. The importance of this experimental finding was emphasized by the detection of numerous naturally occurring ARΔLBD-variants in late stage CRPC cells [28], [36], [37]. Most ARΔLBDs are products of alternative splicing (AR-V) [28], [38], albeit other mechanisms like nonsense-mutations leading to premature chain termination and enzymatic cleavage were also shown to give rise to ARΔLBDs [36], [38], [39]. Although many ARΔLBDs were shown to activate AR-target genes under androgen deprived conditions *in vitro*, they are unable to activate the full panel of AR-dependent genes [4], [30], [31], [36], [38], [40]. While *in vivo* both, the AR and ARΔLBD are expressed in CRPC cells, it was suggested that ARΔLBD- receptors must act in concert with full length AR to activate AR-dependent genes in CRPC [41]. Although there is experimental evidence that ARΔLBDs like AR^v567es^ and Q640X form heterodimers [30], [31] with full length AR *in vitro*, the functional relationships between the AR and ARΔLBD remain largely unknown [41], [42]. However, based on clinical and experimental observations there is no doubt that ARΔLBDs are involved in the progression of CRPC [3], [10]–[12]. With the emergence ARΔLBD in late stage CRPC, all current therapies targeting the AR-LBD either by reducing ligands/androgens or directly with anti-androgens are prone to fail. In consequence, there is an urgent need for novel inhibitors targeting functional domains present in both AR and ARΔLBD receptor types.

To date various studies focused on the isolation of small molecule inhibitors, able to destabilize AR-molecules [43], [44] or to inhibit AR-signalling in a LBD-independent manner [45], [46]. The most promising compounds are those targeting the N-terminal or transactivation domain (NTD/TAD) of the AR [47]–[49]. Unfortunately, the identification of novel drugs targeting the AR-N-terminus is hampered by the high flexibility and intrinsic disorder of the AR-NTD/TAD making it impossible to use a structure based drug design [50]. Instead, time consuming cell based screening assays are used to test each putative compound empirically.

Resveratrol (RSV), a natural stilbene found in grapes and wine is characterized by a broad range of anticancer, antioxidant, anti-inflammatory and cardioprotective activities [13]. Various RSV-analogues have been shown to affect PC formation, growth and metastasis [18], [23]. Besides its ability to inhibit WNT/β-catenin-signalling, an important pathway in PC, RSV has been identified as a potent inhibitor of AR-signalling [16], [17], [24]. Here we analyzed the ability of the naturally occurring RSV and synthetic stilbene FIDAS to impair AR and most important ARΔLBD-signalling in human PC cells.

FIDAS was selected because of its relatively long half life *in vivo* (half lifes: RSV 8–14 min, FIDAS 351 min) [19], [51]. As depicted by reporter gene assays, FIDAS and RSV were able to diminish signalling of the endogenous AR expressed in 22Rv1 and LNCaP cells. To our knowledge this is the first demonstration that the synthetic RSV-analogue FIDAS possesses anti-AR properties.

Several studies suggested that a RSV-induced decrease of AR-mRNA is the predominant mechanism responsible for the anti-androgenic effects of RSV [17], [24]. However, Harada et al. reported that the inhibitory effects of RSV on AR-function are partly attributable to down regulation of the AR at a posttranslational level [14]. Unfortunately, all studies are hampered by the fact that the data were obtained from LNCaP experiments only. In a series of qRT-PCR experiments we were able to demonstrate that in LNCaP cells RSV diminishes not only AR mRNA but also endogenous ARΔLBD mRNA-levels (AR-V7). The second AR-positive PC line 22Rv1 almost dose-dependently down regulated AR and AR-V7 levels following RSV treatment. The second stilbene, FIDAS was able to diminish AR and AR-V7 mRNA in LNCaP but was unable to do so in 22Rv1 cells. This discrepancy prompted us to analyze the effects of FIDAS and RSV on AR and ARΔLBD protein levels. As expected, AR-protein levels were reduced in LNCaP cells following FIDAS-treatment, a finding that is in agreement with the mRNA-data. However, in 22Rv1 cells both compounds increased rather than decreased AR-protein levels, irrespective of previous mRNA-levels. The reason why RSV or FIDAS contribute to a stabilization of the AR-proteins in 22Rv1 cells remains unknown. Although the AR in 22Rv1 cells was shown to be hormone responsive [52]–[54] the receptor molecule presents a duplication of exon 3, thereby generating an AR with a third zinc-finger motif (AR^Ex3dup^) [2]. Albeit no direct interaction between AR^Ex3dup^ and AR-V7 has been shown so far [29] its presence could indirectly affect AR-V7 stability. In PC-3 cells transiently transfected with an AR-expression vector under control of a constitutive SV40 promoter instead of the natural AR-promoter, the AR-protein levels remained unchanged following RSV or FIDAS treatment. These findings are in agreement with similar results observed in AR(+) cells i.e. Hela cells stably transfected with a cytomegalovirus (CMV) promoter driven AR-expression plasmid [17]. The results generated in PC-3 and AR(+) cells suggest that AR-protein levels and/or AR-stability are not affected through posttranslational modifications in all cells.

In order to analyze the effects of FIDAS and RSV on AR/ARΔLBD-signalling, experiments were performed in PC-3 cells, transiently transfected either with AR and/or Q640X, an ARΔLBD recently isolated from a metastatic castration resistant tumor [36]. By using this experimental approach we were able to discriminate the direct effects of FIDAS and RSV on AR and ARΔLBD-proteins from interference due to changes in AR/ARΔLBD-mRNA levels. Interestingly, RSV and FIDAS were able to inhibit transcriptional activity of both AR and Q640X in a dose dependent manner. Down regulation of AR and Q640X-activity for both substances was already significant at a concentration of 50 µM with FIDAS being the more potent inhibitor. The fact that Q640X was inhibited by FIDAS and RSV strongly suggests that stilbenes are AR-inhibitors that are not interfering with the AR-LBD. This assumption is furthermore supported by a previous finding of Harada et al. showing that RSV decreased the transcriptional activity of ARΔC-Nuc, an artificially truncated, constitutively active AR-variant (amino acids 1–660) [14]. While *in vivo* AR and ARΔLBD are concomitantly expressed it was hypothesized that under androgen deprived conditions, AR and ARΔLBD must form transcriptionally active AR/ARΔLBD-heterodimers in order to activate AR-dependent genes [41]. In agreement with this hypothesis, we recently demonstrated that under specific experimental conditions (concentration AR>Q640X) co-expression of AR and Q640X resulted in the formation of AR/Q640X-heterodimers characterized by a significant activation of a PSA-promoter plasmid in the absence of androgens [30]. Using this experimental approach we were able to show that RSV and FIDAS reduced the AR/Q640X- PSA-reporter gene activity by 61% and 71%, respectively.

The decrease of AR and Q640X-signalling following stilbene treatment was not due to an inhibition of nuclear translocation as shown by fluorescence microscopy of green fluorescent AR or Q640X fusion proteins transfected into PC-3 cells. Although these findings differ from a study analyzing AR nuclear translocation in LNCaP cells [16] they are in agreement with the data recently published by Shi et al. [17].

In order to generate genomic signals the AR and Q640X must form receptor dimers in the nucleus [30], [32]. The ability of FIDAS and RSV to inhibit AR/Q640X-heterodimer-induced signals strongly recommended the analysis of AR and/or Q640X dimerization processes [30]. To our surprise RSV as well as FIDAS induced a strong decrease of AR and Q640X homodimerization as well as AR/Q640X heterodimerization. To our knowledge this is the first report to show that stilbenes are able to inhibit the formation of all forms of AR or ARΔLBD-dimers. The inhibition of AR-dimerization seen in RSV or FIDAS treated cells could explain the disruption in AR-DNA-binding recently described by Harada et al. [16]. Interestingly, the decrease in DNA binding was preceded by a decrease in AR-acetylation following RSV-treatment [16]. As acetylation processes are affecting stability, heterodimerization, DNA-binding and transactivation of FXR, another member of the steroid receptor superfamily [55] it is tempting to speculate that the inhibition of AR-dimerization could be linked to stilbene modulated acetylation.

Unfortunately, the concentrations of FIDAS and RSV, necessary to inhibit AR-dimerization are relatively high. Results from pharmacokinetic studies indicate that circulating stilbenes are rapidly metabolized in the body and raise doubts on the physiological relevance of the concentrations typically used for *in vitro* experiments. Using a modified chorioallantoic membrane xenograft model we proved that even low systemic concentrations RSV and FIDAS were able to inhibit the proliferation of PC cells. Interestingly, the inhibition of proliferation was the most pronounced in AR-positive cells and paralleled by a down regulation of PSA in the tumor xenografts. Although numerous clinical data are available on the safety of RSV as a dietary supplement (www.clinicaltrials.gov), the effects of a long term treatment with high doses of RSV remain to be elucidated. Moreover, the pharmacokinetics, bioavailability, and toxicity of synthetic RSV-derivatives like FIDAS, generated to improve the low bioavailabilty of natural stilbenes, need to be determined.

The effects of stilbenes on the AR and androgen-responsive genes are complex. However, gene expression data suggest that the down-regulation of androgen-responsive genes is not solely due to decreased levels of the AR [56]. To our knowledge this is the first report showing that stilbenes like RSV and FIDAS are able to inhibit the dimerization of different AR-variants. Although the precise mechanisms underlying this phenomenom remain largely unknown, a more profound analysis of this interesting pharmacological feature, could lead to the development of more potent inhibitors of AR-dimerization.

## Supporting Information

Figure S1 **Representative control experiments for the M2H.** PC-3 cells were transiently transfected with pAR-VP16/ACT (pAR-ACT), pAR-GAL4/BIND (pAR-BIND), pQ640X-VP16/ACT (pQ640X-ACT), pQ640X-GAL4/BIND (pQ640X-BIND) or the empty vectors pACT and p-BIND as shown in Figure S1 (A–C). Dimer formation was analyzed after 24 hours in the presence/absence of androgens using the M2H-Assay described in Material and Methods. (A) Control experiments for AR-homodimerization in the absence of androgens. (B). Control experiments for AR-homodimerization in the presence of androgens. (C) Control experiments for Q640X-homodimerization. Data shown in A–C represent the mean of 3 independent experiments. Results are expressed in % basal activity of the M2H-reporter co-transfected with pAR-GAL4/BIND and pAR-VP16/ACT in the absence of androgenic stimuli ( = 100%, right bar).
(TIF)

Table S1 **Effects of RSV and FIDAS on the cell viability** ***in vitro*** **.**
(DOC)
