# Supplementary figures and images for: Stilbene Induced Inhibition of Androgen Receptor Dimerization: Implications for AR and ARΔLBD-Signalling in Human Prostate Cancer Cells
Source: PLoS One. 2014 Jun 2;9(6):e98566. doi: 10.1371/journal.pone.0098566 (PMC4041728; doi:10.1371/journal.pone.0098566)

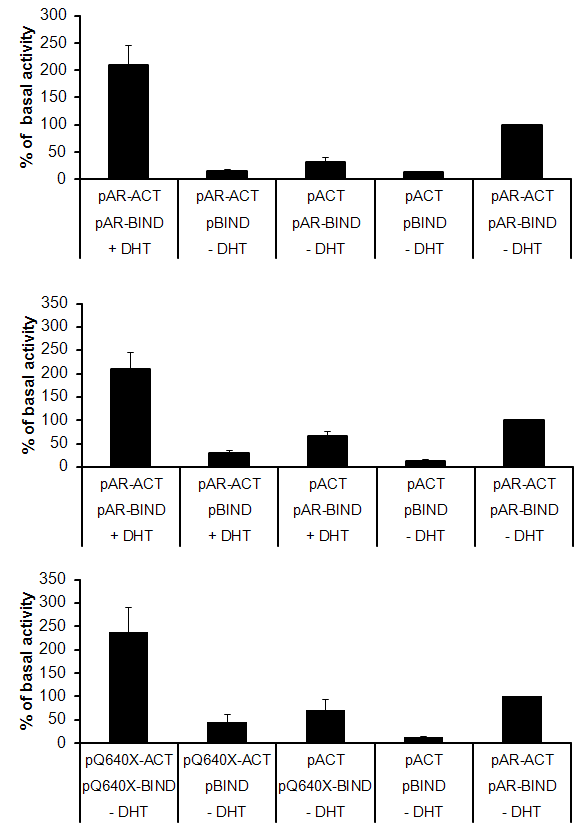

Supplement: Figure S1 — Representative control experiments for the M2H. PC-3 cells were transiently transfected with pAR-VP16/ACT (pAR-ACT), pAR-GAL4/BIND (pAR-BIND), pQ640X-VP16/ACT (pQ640X-ACT), pQ640X-GAL4/BIND (pQ640X-BIND) or the empty vectors pACT and p-BIND as shown in Figure S1 (A–C). Dimer formation was analyzed after 24 hours in the presence/absence of androgens using the M2H-Assay described in Material and Methods. (A) Control experiments for AR-homodimerization in the absence of androgens. (B). Control experiments for AR-homodimerization in the presence of androgens. (C) Control experiments for Q640X-homodimerization. Data shown in A–C represent the mean of 3 independent experiments. Results are expressed in % basal activity of the M2H-reporter co-transfected with pAR-GAL4/BIND and pAR-VP16/ACT in the absence of androgenic stimuli ( = 100%, right bar). (TIF) [file pone.0098566.s001.tif]
