# Supplementary material for: Stilbene Induced Inhibition of Androgen Receptor Dimerization: Implications for AR and ARΔLBD-Signalling in Human Prostate Cancer Cells
Source: PLoS One. 2014 Jun 2;9(6):e98566. doi: 10.1371/journal.pone.0098566 (PMC4041728; doi:10.1371/journal.pone.0098566)
Supplement: Table S1 — Effects of RSV and FIDAS on the cell viability in vitro . (DOC) [file pone.0098566.s002.doc]

**Table 1S.** Effects of RSV and FIDAS on the cell viability *in vitro*

|  |  | **RSV** | | **FIDAS** |
| --- | --- | --- | --- | --- |
|  | **0 µM** | **50 µM** | **100 µM** | **50 µM** |
| **PC-3** | 100,0 | 89,2 ± 7,7 | 71,9 ± 7,1 | 119,1 ± 14,7 |
| **22Rv1** | 100,0 | 93,1 ± 6,9 | 84,5 ± 8,6 | 113,0 ± 16,8 |
| **LNCaP** | 100,0 | 88,9 ± 23,5 | 85,5 ± 20,4 | 126,1 ± 12,3 |

Cell viability of AR positive (22Rv1, LNCaP) and AR-negative (PC-3) prostate cancer cells treated with RSV and FIDAS for 24 hours was assessed by means of a colorimetric MTT-assay measuring the reduction of tetrazolium salts to formazan derivatives by functional mitochondria (Mossmann T. (1983), J Immunol Methods 65: 55–62). Within this time frame RSV as well as FIDAS did not exhibit a significant *in vitro* toxicicity interfering with the dual luciferase based reportergene or the M2H assays.
